# Case Report: A case of right ventricular metastasis from cervical cancer presenting with thrombocytopenia: the role of echocardiography and myocardial contrast echocardiography

**DOI:** 10.3389/fonc.2026.1774949

**Published:** 2026-03-05

**Authors:** Qian Yang, Dong Bai, Liming Bao, Lei Liang, Haijun Hou

**Affiliations:** 1 Department of Ultrasound, Aerospace Center Hospital, Beijing, China; 2 Department of Radiology, Aerospace Center Hospital, Beijing, China; 3 Department of Cardiac Surgery, Aerospace Center Hospital, Beijing, China

**Keywords:** echocardiography, heart neoplasms, myocardial contrast echocardiography, neoplasm metastasis, uterine cervical neoplasms

## Abstract

**Background:**

Cardiac metastasis from cervical cancer is rare and often presents with nonspecific symptoms, leading to diagnostic delays. This case highlights the role of myocardial contrast echocardiography (MCE) in detecting such metastases in long-term cervical cancer survivors.

**Case presentation:**

A 63-year-old female with a history of cervical cancer treated 11 years ago presented with thrombocytopenia and respiratory symptoms. Imaging revealed a mobile mass in the right ventricle extending into the pulmonary artery. MCE showed peripheral rim enhancement, indicative of a necrotic malignant tumor, confirmed as metastatic squamous cell carcinoma.

**Therapeutic intervention:**

The patient underwent surgical resection of the right ventricular mass and tricuspid valvuloplasty. Her thrombocytopenia resolved post-surgery, and no further oncological treatment was needed.

**Conclusion:**

MCE is a valuable tool for diagnosing cardiac metastases, especially in cervical cancer survivors. This case underscores the need for long-term follow-up and imaging surveillance due to the risk of delayed and atypical metastasis.

## Introduction

Cervical cancer continues to pose a significant global health burden, with approximately 500,000 new cases diagnosed annually worldwide (1). For early-stage lesions, such as microinvasive carcinomas, the prognosis is generally favorable following standard surgical treatment, with low recurrence rates. Copeland et al. (2) conducted a retrospective analysis of 180 patients with squamous cell cervical carcinoma (invasion depth ≤5 mm) and found a pelvic lymph node metastasis rate of only 1%, with no mortality associated with recurrence in the cohort. The most frequent sites of distant metastasis in cervical cancer include the lungs, liver, and bones (3). In contrast, cardiac metastasis from cervical cancer remains extremely rare, a finding supported by Simek et al. (4) This case highlights that while echocardiography is the primary screening tool for cardiac masses, contrast-enhanced echocardiography offers significant incremental value in differential diagnosis by characterizing tumor vascularization.

For patients with cervical cancer, including those with early-stage disease, long-term follow-up remains essential. Distant metastases may occur more than a decade after radical surgery, and clinical presentations are often atypical and nonspecific, necessitating a high index of clinical suspicion as well as appropriate imaging evaluation.

## Methods

### Study design and participant

This case report presents the case of a 63-year-old female patient who was admitted to our hospital with thrombocytopenia (platelet count 60 × 10^9^/L). She had a history of cervical cancer, treated with transvaginal total hysterectomy 11 years earlier. She has no known drug allergies, and no significant family or genetic predispositions were noted. Postoperative pathology revealed cervical squamous intraepithelial neoplasia (CIN) II-III with focal early invasion (depth 0.1 cm). Informed consent was obtained from the patient for the publication of this case report. She does not report any significant psychosocial stressors or mental health conditions. Postoperatively, she had an uneventful recovery and was followed up regularly for 11 years, with no signs of recurrence or metastasis.

### Diagnostic evaluations

Upon admission, a comprehensive assessment was performed. Initial blood tests revealed thrombocytopenia, with normal white blood cell count and hemoglobin levels. Cardiac evaluation included transthoracic echocardiography, which identified a mass in the right ventricle. Further imaging with CTPA and MCE was performed to characterize the mass. Transthoracic echocardiography was performed using standard protocols, and MCE employed microbubble agents to assess myocardial perfusion.

The patient presented with thrombocytopenia and respiratory symptoms, including intermittent cough, blood-streaked sputum, chest tightness, and exertional dyspnea. No fever or chest pain was reported.

### Intervention

After multidisciplinary review, the patient underwent resection of the ventricular mass and tricuspid valvuloplasty. The procedure aimed to obtain a definitive histological diagnosis and alleviate potential obstruction. Percutaneous endomyocardial biopsy was not performed because the patient had an urgent surgical indication due to pulmonary embolism and the risk of intracardiac obstruction. Surgical resection was therefore chosen to both relieve hemodynamic compromise and obtain definitive histopathological diagnosis. Therapeutic intervention involved the surgical resection of the right ventricular mass and tricuspid valvuloplasty. The decision for surgery was based on imaging findings and the isolated nature of the cardiac metastasis. Given the lack of evidence for metastasis in other organs, as confirmed by comprehensive imaging work-up, no additional chemotherapy or radiotherapy was administered.

### Ethical considerations

The study was conducted in accordance with institutional ethical guidelines, and patient anonymity was strictly maintained.

## Results

### Clinical presentation

The patient presented with thrombocytopenia and respiratory symptoms, including intermittent cough, blood-streaked sputum, chest tightness, and exertional dyspnea. No fever or chest pain was reported.

### Imaging findings

**Figure 1:**
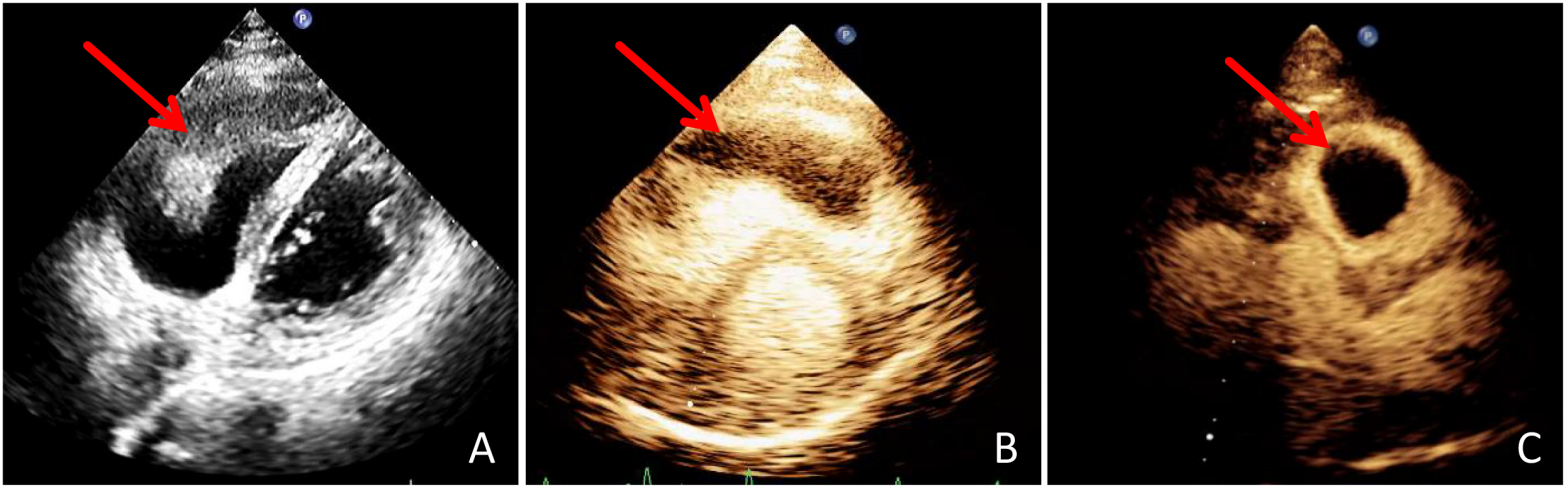
Echocardiography **(A)** reveals an isoechoic mass within the right ventricular cavity that is connected to the right ventricular wall and extends into the pulmonary artery(arrow). MCE **(B)** shows no significant contrast filling within the mass in the right ventricular cavity (arrow). After rotating the probe 90 degrees, the cross-sectional view **(C)** demonstrates no significant contrast filling within the mass in the right ventricular cavity (arrow).

**Figure 2:**
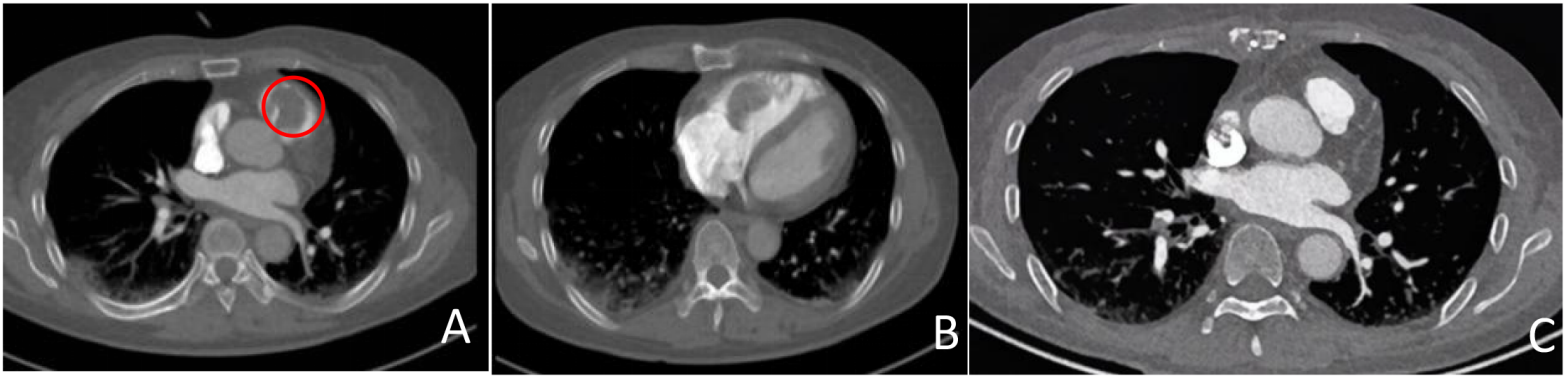
CTPA **(A, B)** reveals a filling defect within the lumen of the right ventricle (circle). The interface between this lesion and the right ventricular wall is indistinct. After the operation, the filling defect of the right ventricle was not clearly displayed **(C)**.

Echocardiography demonstrated a mobile, isoechoic intracavitary mass (4.6 cm × 2.2 cm) within the right ventricle, attached to the lateral wall and extending into the pulmonary artery, associated with elevated pulmonary artery systolic pressure (54 mmHg). MCE revealed an irregular mass measuring approximately 6.1 cm × 2.2 cm, characterized by peripheral rim enhancement with absence of internal perfusion, indicating central necrosis (**Figure 1**). CTPA confirmed filling defects in the pulmonary artery and an ill-defined mass in the right ventricle (**Figure 2**).

### Histopathological and laboratory results

**Figure 3:**
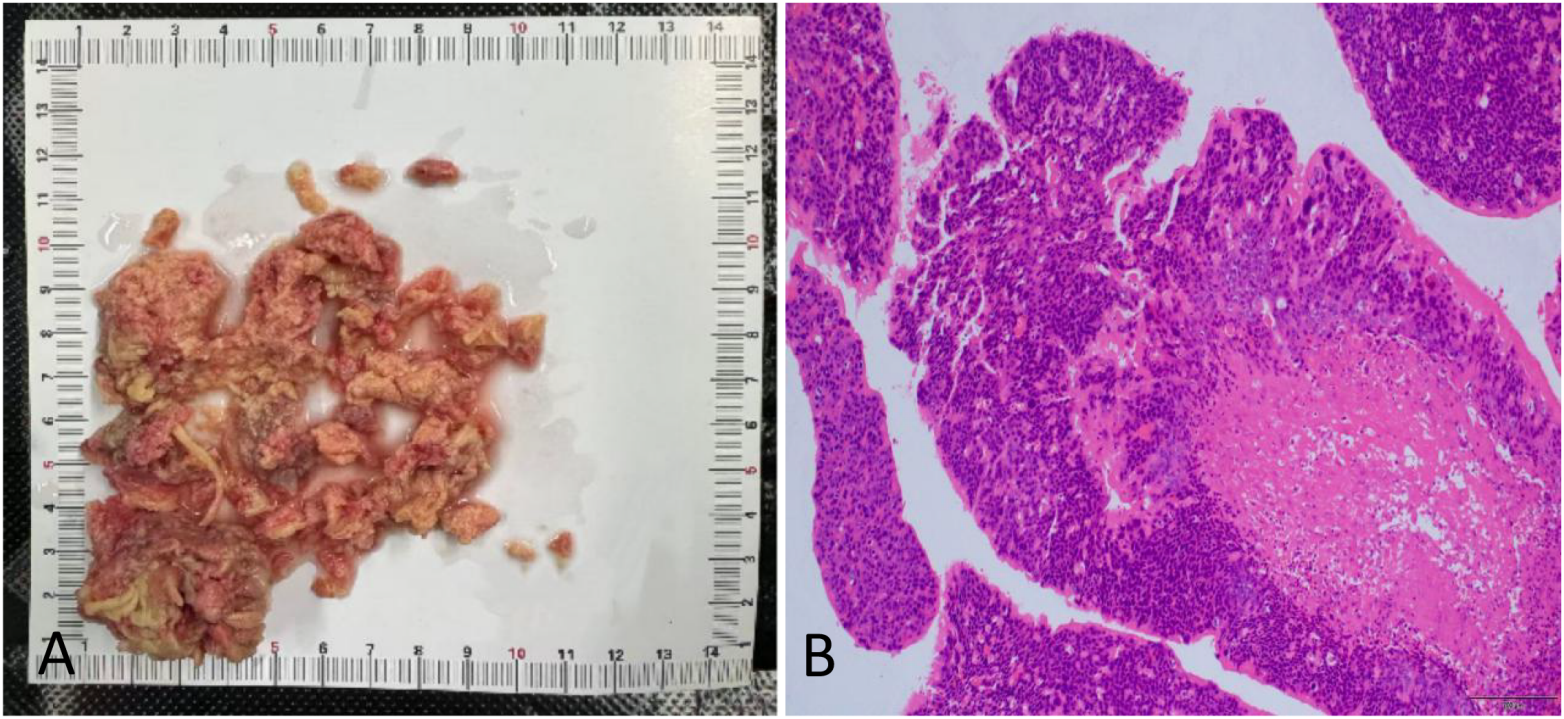
Intraoperatively, the resected mass fragment from the right ventricle exhibited a loose texture **(A)**. Pathological section of the right ventricular mass at a magnification of ×10 **(B)**.

Postoperative pathology confirmed moderately to poorly differentiated squamous cell carcinoma in the right ventricle. In the context of the patient’s medical history and immunohistochemical findings, the lesion was diagnosed as metastatic cervical carcinoma. Immunohistochemistry showed CK7 (+/−), P40 (−), P16 (−), a high Ki-67 proliferation index (~80%), and PD-L1 expression with a combined positive score <1 (**Figure 3**). Tumor markers, including squamous cell carcinoma antigen and neuron-specific enolase, were elevated preoperatively.

### Follow-up

**Figure 4:**
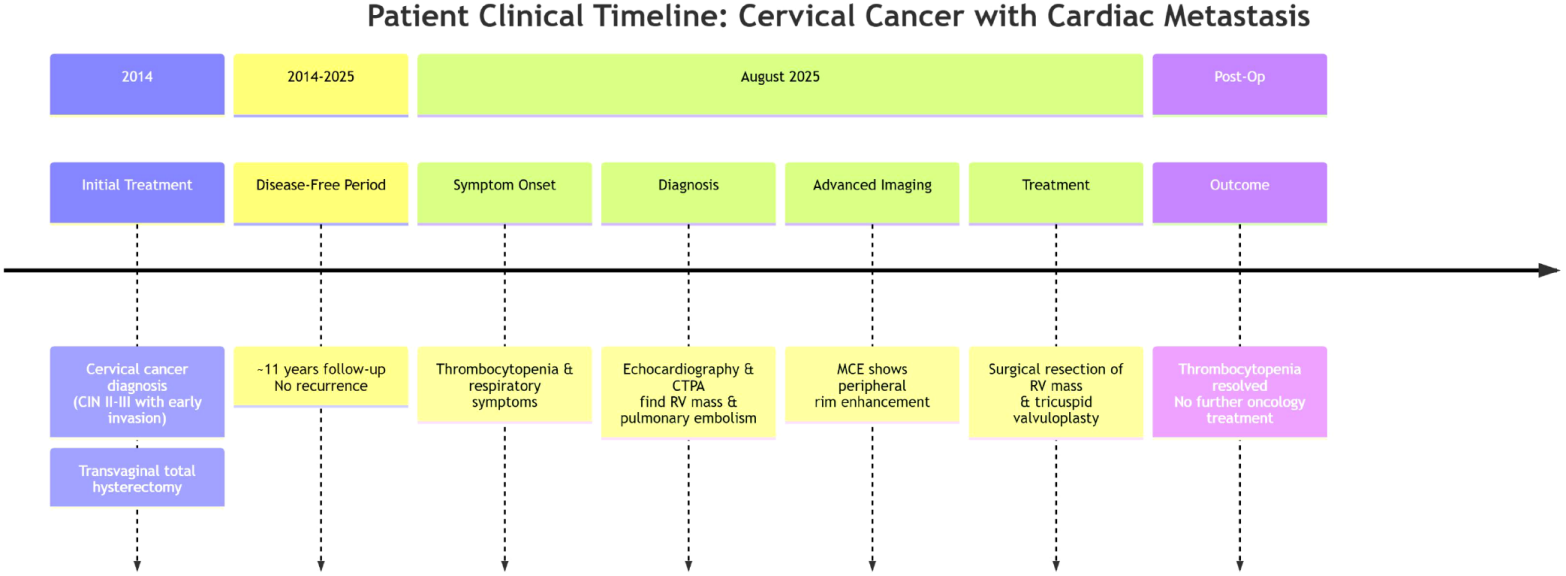
This timeline illustrates the clinical course of a patient with a rare case of cardiac metastasis from cervical cancer. It details key events from the initial diagnosis and surgical treatment in 2014, an 11-year disease-free period, through the presentation of symptoms, diagnostic work-up, and definitive surgical intervention in August 2025. The color-coded, horizontal flow design provides a clear visual summary of the diagnostic journey and critical decision points.

The patient underwent surgery to resect the right ventricular mass, with concurrent tricuspid valvuloplasty to address any associated hemodynamic concerns. The postoperative recovery was uncomplicated, and her thrombocytopenia resolved. She showed no signs of cardiac dysfunction and recovered without incident. Given the isolated nature of the metastasis and the absence of extracardiac involvement, as confirmed by imaging, no further oncological treatments, such as chemotherapy or radiotherapy, were deemed necessary (**Figure 4**).

## Discussion

Cervical cancer continues to impose a substantial global health burden, particularly among younger populations, while prevention, screening, and treatment strategies remain suboptimal in many regions (5). Hematogenous metastasis, although less frequent than lymphatic spread, is associated with higher mortality, with the lungs and bones being the most commonly affected sites (6). However, cardiac involvement remains exceptionally rare in the medical literature (7). This case is notable for several reasons, including the early stage of the primary tumor, the unusually long interval of 11 years between initial treatment and recurrence, and metastasis to a highly uncommon site—the heart. These features underscore the need for prolonged clinical vigilance and extended follow-up strategies, even in patients initially diagnosed with early-stage disease. Despite the generally poor prognosis associated with cardiac metastasis, surgical resection was performed due to the patient’s presentation with pulmonary embolism and right ventricular outflow obstruction, which constituted an emergency indication for intervention. Additionally, the long disease-free interval and the early-stage nature of the primary cervical cancer supported an aggressive therapeutic approach aimed at alleviating symptoms and prolonging survival.

To exclude extracardiac metastases, a comprehensive imaging work-up was conducted, including chest, abdominal, and pelvic CT scans, cranial CT, and pelvic MRI with contrast. These imaging modalities revealed no evidence of metastases in other organs, confirming that the right ventricular metastasis was isolated. Given the absence of symptoms indicative of systemic metastasis, a PET-CT scan was not performed in this case. The decision to proceed with surgical resection of the metastatic mass, followed by tricuspid valvuloplasty, was based on the rarity of cardiac metastasis and the isolated nature of the lesion. The lack of symptoms suggesting systemic metastasis, alongside a thorough imaging evaluation, led us to refrain from pursuing chemotherapy or radiotherapy, which are typically considered in cases of widespread metastasis.

Notably, thrombocytopenia was the initial and predominant clinical manifestation in this patient, leading to diagnostic delay and inappropriate management. She was repeatedly referred to hematology departments across multiple institutions and treated for presumed primary hematologic disorders without significant improvement. Importantly, normalization of the platelet count following surgical resection of the right ventricular mass strongly suggests a paraneoplastic mechanism. Throughout the clinical course, the patient remained largely asymptomatic from a cardiac perspective, with preserved cardiac function.

Intracardiac masses encompass a broad differential diagnosis, including benign and malignant primary tumors, metastatic lesions, direct invasion from adjacent malignancies, and non-neoplastic mimics such as thrombi or vegetations (8). Although primary cardiac tumors are rare, metastatic cardiac involvement is far more common (9). Accurate differentiation among these entities is essential, as management strategies differ substantially: myxomas typically require surgical excision, whereas thrombi are generally managed with anticoagulation. Therefore, establishing a systematic imaging-based diagnostic approach is critical for optimal clinical decision-making.

Transthoracic echocardiography is the first-line imaging modality for suspected cardiac masses owing to its wide availability, high temporal resolution, and ability to assess lesion mobility and hemodynamic impact (8). MCE further enhances diagnostic accuracy by providing functional information regarding tissue perfusion and vascularity (10–12). In general, thrombi show no contrast enhancement, whereas benign tumors such as myxomas typically demonstrate homogeneous or partial enhancement (13, 14). In the present case, the absence of internal enhancement mimicked a thrombus; however, the presence of peripheral rim enhancement suggested neovascularization at the tumor margin, a characteristic feature of malignant tumors with central necrosis. This imaging pattern provided an important clue to the malignant nature of the lesion prior to histopathological confirmation.

This case underscores the rarity of cardiac metastasis in cervical cancer and demonstrates the effective use of advanced imaging techniques, such as MCE and echocardiography, which enabled early detection and informed treatment decisions. However, this single-case report limits the generalizability of the findings. Larger studies are needed to validate the role of MCE and echocardiography in detecting cardiac metastasis. Although PET-CT was not performed, its inclusion could have provided a more comprehensive evaluation of potential extracardiac metastases. Additionally, the relatively short follow-up period emphasizes the need for long-term surveillance to monitor for recurrence or additional metastasis.

This case highlights the pivotal role of echocardiography, particularly MCE, in the detection and characterization of intracardiac metastases. For patients with cervical cancer, including those with early-stage disease, long-term follow-up and a high index of clinical suspicion are essential. Timely imaging evaluation should be considered when unexplained systemic manifestations occur, given the potential for atypical and delayed metastatic presentations.

## Data Availability

The original contributions presented in the study are included in the article/supplementary material. Further inquiries can be directed to the corresponding author.
